# The Effects of Pin Profile on HDPE Thermomechanical Phenomena during FSW

**DOI:** 10.3390/polym14214632

**Published:** 2022-10-31

**Authors:** Hassanein I. Khalaf, Raheem Al-Sabur, Murat Demiral, Jacek Tomków, Jerzy Łabanowski, Mahmoud E. Abdullah, Hamed Aghajani Derazkola

**Affiliations:** 1 Mechanical Department, Engineering College, University of Basrah, Basrah 6100, Iraq; 2 College of Engineering and Technology, American University of the Middle East, Egaila 54200, Kuwait; 3 Institute of Manufacturing and Materials Technology, Faculty of Mechanical Engineering and Ship Technology, Gdańsk University of Technology, Gabriela Narutowicza Street 11/12, 80-233 Gdańsk, Poland; 4 Mechanical Department, Faculty of Technology and Education, Beni-Suef University, Beni-Suef 62511, Egypt; 5 Department of Mechanics, Design and Industrial Management, University of Deusto, Avda Universidades 24, 48007 Bilbao, Spain

**Keywords:** friction stir welding, high-density polyethylene (HDPE), thermomechanical modeling, thermal study, materials flow

## Abstract

Friction stir welding (FSW) of polymeric materials has recently attracted significant attention. Herein, we present the effect of the tool pin profile on the FSW of high-density polyethylene (HDPE) joints through joint experimental analysis and thermomechanical simulations. For analysis of pin profile effects on the thermomechanical properties of HDPE joints, frustum (FPT), cubic (CPT), and triangular (TPT) pin shapes were selected in this study. This research investigated the heat generation of the parts of the different tools as well as heat flux (internal and surface). The results revealed that the heat generation in pins with more edges (cubic (96 °C) and triangular (94 °C)) was greater than in pins with a smooth shape (frustum (91 °C)). The higher heat generation caused the heat flux on the surface of the HDPE from the cubic pin profile to be greater than for other joints. Due to the properties of HDPE, higher heat generation caused higher material velocity in the stirring zone, where the velocity of the materials in TPT, CPT, and FPT pins were 0.41 m/s, 0.42 m/s, and 0.4 m/s, respectively. The simulation results show sharp-edged pins, such as triangular and cubic, lead to over-stirring action and internal voids formed along the joint line. Furthermore, the simulation results indicated that the size of the stirred zones (SZs) of the FPT, TPT, and CPT samples were 17 mm^2^, 19 mm^2^, and 21 mm^2^, respectively, which is around three times the corresponding values in the HAZ.

## 1. Introduction

The ability to join non-weldable materials is considered one of the main benefits of friction stir welding (FSW). In addition, this ability extends to achieving high-quality joints of similar and dissimilar materials, either metallic or non-metallic, where it has become a revolutionary welding technology [1,2]. In the FSW process, the joint quality is affected by several parameters, primarily the rotational speed of the tool and its traverse velocity. In contrast, other parameters such as tilt angle, dwelling time, tool geometry, and applied load have less impact [3]. The importance of studying the pin profile is due to its direct influence on the metal flow in the stirred zone (SZ) and the variation in the heat generated, which determines the mechanical properties of the welded joint [4]. Mugada and Adepu [5] investigated the effects of different tool pin profiles on the FSW of 6082 aluminum alloy. They used tapered cylindrical, hexagonal, square, pentagonal, and triangular tool pin profiles. They indicated that due to the pulsating (shearing) action of the base metal, the initial force for the plunging phase reduces at FSW with polygonal pins compared to the tapered cylindrical pin. During the overall FSW process, a tapered cylindrical pin gave a constant stability force compared to other pins.

Elangovan et al. [6] investigated the influence of tool pin profiles on the properties of FSWed AA6061 alloy. They reported on FSW using polygonal pins and allowing the aluminum alloy to pass around the pin profile. Accordingly, the polygonal pin profile led to better joint properties. They investigated the pin profile’s effect on the FSWed sample’s mechanical properties. The results showed that the joints produced with the square pin profile had better mechanical properties than the other pin shapes.

Biswas et al. [7] revealed that FSW tool pin geometry plays a crucial role in material deformation, but the pin’s generated heat is relatively affected by the final properties of the welded samples. Khodaverdizadeh et al. [8] investigated threaded and square tool pin effects on the FSW of copper. They reported that finer microstructures form in the stirring zone (SZ) due to the edges’ eccentric action in the square pin profile. Imam et al. [9] investigated different tool pin profiles during the FSW of AA6063 aluminum alloys. They showed that tapered pins increased the possibility of defect formation in joint lines.

Mehta et al. [10] investigated the adhesion of base material on polygonal FSW tool pin profiles. They found that the permanent adhesion of plasticized material on pin sides decreases at higher tool rotational velocity—the applied stress on the tool pin decreased with increasing numbers of pin sides. A higher number of pin sides increases the rate of frictional heat generation [11]. Rao et al. [12] experimented on FSW of AA2219 aluminum alloy with conical and triangular pin profiles. They discovered that the microstructure of the SZ formed with a triangular pin profile had more refined grains than the sample FSWed with a conical tool pin. According to their results, the geometry of the pin profile can intensely affect the properties of the final joint. Amirafshar and Poraliakbar [13] reported that finer grain size was attained with a square pin by comparing with triangular, cylindrical, and conical pin profiles.

The FSW process freshly joins polymeric materials and metals to produce lightweight structures [14]. Due to the sensitivity of these materials, finding process parameters like pin profiles to decrease internal defects are significant. Heat generation during the process of FSW of polymeric materials is critical to the final product’s quality [15].

Despite the existing literature on the pin profile’s role in the FSW of metallic base materials, few studies on polymers during FSW have been reported. Material flow investigation during FSW of PP revealed that a threaded frustum pin tool yielded higher joint shear strength and better surface quality due to controlled material flow in the stirring zone [16]. Using an inappropriate tool leads to the formation of pores, cavities, lack of consolidation, and inclusions in the joint line. The considerable shear force and high peak temperature significantly influence void formation, affect mechanical properties, and lead to complex behaviors for material flow [17].

Researchers show that the thread design and rotational direction also affect nylon 6 FSW joint strength and material flow [18], and the shear strength of PC polymer after FSW significantly varied with different shoulder diameters [19]. This result indicates that the FSW tool shoulder has an essential role in final joint properties [20]. Furthermore, a non-contact shoulder tool gives a smaller formed zone than regular polymer joints [21].

The FSW tool pin’s shape is also an essential factor affecting polymeric material flow [22]. Hajideh et al. [23] investigated the effect of pin profiles on FSW of a dissimilar joint between PE and PP. They used triangular, square, threaded, and straight cylindrical pin profiles to evaluate the final joint properties. They showed that the threaded pin profile improved the mechanical properties of the final joint. The squared and triangular pin profiles severely stirring the plasticized material at the SZ. Sahu et al. [24] indicated that a cylindrical pin produces a sound PP joint after FSW, and the square pin profile creates high fluidity and throws material out of weld lines. Sadeghian and Besharati Givi [25] stated that during ABS friction stir joining, the material stirring of the cylindrical pin tool was less uniform than that of a conical pin. Aghajani Derazkola and Simchi [26] indicated that during FSW of high crystallinity polymers like PMMA, the edged pin profile increased internal defects in the final joint.

On the other hand, due to the FSW process’s complexity, it is impossible to monitor internal heat generation during the FSW of polymeric materials. For this reason, the researchers used CFD simulation techniques to understand the effects of tool pin profiles on thermal history during the FSW of polymers.

## 2. Modelling of the FSW Process

### 2.1. Description of the Model

ANSYS FLUENT software was used in this research to simulate 3D material flow variation during FSW using different pin profiles. The forward movement of the FSW tool was considered preliminary, while tool exit steps were ignored to decrease simulation time and increase the simulation result’s reliability during simulation. Therefore, a steady-state coupled material flow was utilized for the simulation. The FSW tools and the workpiece dimensions were designed to satisfy the actual dimensions. In the simulation domain, the welding direction was set to the *x*-axis, and the *z*-axis was set as the FSW tool’s normal axis. The *g*, *h*, and *f* signs were used to represent the material velocity in the *x*, *y*, and *z* directions.

In this simulation, the continuity and energy equations were used as below [27,28]:

(1) $$\frac{dg}{dx}+\frac{dh}{dy}+\frac{df}{dz}=0$$

(2) $$\rho c\left(\frac{\partial T}{\partial t}+g\frac{\partial T}{\partial x}+h\frac{\partial T}{\partial y}+f\frac{\partial T}{\partial z}\right)+\dot{H}=k\left(\frac{\partial^{2}T}{\partial x^{2}}+\frac{\partial^{2}T}{\partial y^{2}}+\frac{\partial^{2}T}{\partial z^{2}}\right)$$

For the *x*, *y*, and *z* directions, the momentum equations can be written as below [29]:

(3) $$\frac{\partial g}{\partial t}+g\frac{\partial g}{\partial x}+h\frac{\partial g}{\partial y}+f\frac{\partial g}{\partial z}=F_{x}-\frac{1}{\rho }\frac{\partial P}{\partial x}+\nu \left(\frac{\partial^{2}g}{\partial x^{2}}+\frac{\partial^{2}g}{\partial y^{2}}+\frac{\partial^{2}g}{\partial z^{2}}\right)$$

(4) $$\frac{\partial h}{\partial t}+g\frac{\partial h}{\partial x}+h\frac{\partial h}{\partial y}+f\frac{\partial h}{\partial z}=F_{y}-\frac{1}{\rho }\frac{\partial P}{\partial y}+\nu \left(\frac{\partial^{2}h}{\partial x^{2}}+\frac{\partial^{2}h}{\partial y^{2}}+\frac{\partial^{2}h}{\partial z^{2}}\right)$$

(5) $$\frac{\partial f}{\partial t}+g\frac{\partial f}{\partial x}+h\frac{\partial f}{\partial y}+f\frac{\partial f}{\partial z}=F_{z}-\frac{1}{\rho }\frac{\partial P}{\partial z}+\nu \left(\frac{\partial^{2}f}{\partial x^{2}}+\frac{\partial^{2}f}{\partial y^{2}}+\frac{\partial^{2}f}{\partial z^{2}}\right)$$

**Figure 1:**
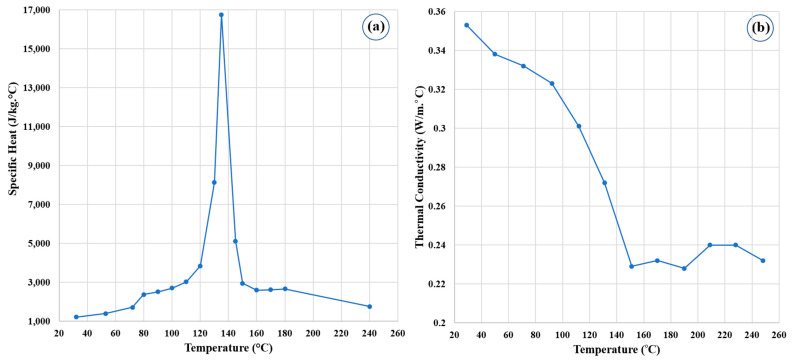
(**a**) Specific heat and (**b**) Thermal conductivity of HDPE.

In this work, the HDPE polymer was assumed as a non-Newtonian single-phase fluid, and the specific heat and thermal conductivity were temperature dependent. As shown in Figure 1a, the specific temperature went up gradually as the temperature increased to 100 °C, then went up sharply to a maximum at about 135 °C, and then went down in a similar behavior. In contrast, the thermal conductivity gradually decreased with the increase in temperature until about 135 °C, as it began to fluctuate around constant values, as shown in Figure 1b. The simulation domain was modelled in ANSYS FLUENT software, and equations were solved using the computational fluid dynamics (CFD) package.

### 2.2. Weld Metal Model

**Figure 2:**
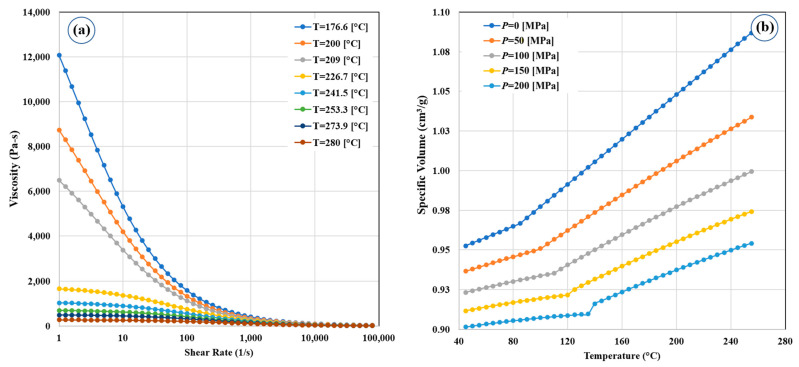
(**a**) Viscosity–shear rate and (**b**) Specific volume of HDPE.

In FSW, the governing equations of the base material properties of the polymers are different from metallic ones because of the different structures of each. In this case, the polymer’s density is defined by specific volume ($\frac{c}{v}$). Temperature and time are the main influencing factors in polymer flow rate study, so the polymers should be warmed up and stirred until they reach a softening state. The time from zero until softening start is known as the “time to flow”. The time to flow as a function of load and temperature of the HDPE polymer is presented in Figure 2a.

The specific volume of HDPE is a temperature and pressure-dependent property [30], and it can usually be represented by the polymer’s pressure–volume–temperature (P–V–T) diagram. For the HDPE used in the current study, the temperature and pressure in the SZ were considered locally because the heat transfer properties of polymeric materials are low. The P–V–T diagram of the HDPE is presented in Figure 2b.

### 2.3. Boundary Conditions

The HDPE plastic deformation heat (*H_p_*), alongside the heat generated by the frictional sliding contact of the tool and polymer interfaces (*H_h_*), represents the total heat generated (H in Equation (2)) during the FSW process [31]:

(6) $$\dot{H}=H_{h}+H_{p}$$

The heat generated by sliding friction at the interfaces of the HDPE with the FSW tool is represented by [32]:

(7) $$H_{h}=\left[\left(1-\delta \right)\chi \tau +\delta \mu_{f}F_{z}\right]\left(\omega r-g\sin\theta \right)$$

The radial distance from the axis of the FSW tool is represented by (*r*) in Equation (7), while *θ* is the FSW pin angle. *τ* demonstrates the polymer shear stress. The *δ* is the fractional slip between the FSW tool and the polymer, *χ* is the coefficient for mechanical efficiency, the friction coefficient is represented by µf, *ω* is the FSW rotational speed, and *F_z_* is the force that is axially exerted on the tool. The amount of heat that is generated by plastic deformation (*H_p_*) is calculated by [33]:

(8) $$H_{p}=\psi \left(\frac{\partial P}{\partial x}+\frac{\partial P}{\partial y}+\frac{\partial P}{\partial z}\right)\left[\begin{array}{c} 2\left({\left(\frac{\partial g}{\partial x}\right)}^{2}+{\left(\frac{\partial h}{\partial y}\right)}^{2}+{\left(\frac{\partial f}{\partial z}\right)}^{2}\right)\\ +{\left(\frac{\partial g}{\partial y}+\frac{\partial h}{\partial x}\right)}^{2}+{\left(\frac{\partial g}{\partial z}+\frac{\partial f}{\partial x}\right)}^{2}+{\left(\frac{\partial f}{\partial y}+\frac{\partial h}{\partial z}\right)}^{2} \end{array}\right]$$

where 𝜓 is the internal mixing factor in the stirring zone.

### 2.4. Heat Transfer Model

Regarding the simulation of heat transfer during the FSW process, heat transfer conditions are different according to the contact regions between the HDPE workpiece and the FSW tool (conduction), as well as exposure of the HDPE workpiece and tool to the environment (convection and radiation). In this study, several heat transfer equations were used. The backing plate and the bottom of the HDPE were in direct contact. Accordingly, conductive heat transfer was considered at the bottom surface of the HDPE and the welding fixture [34]:

(9) $$k{\frac{\partial T}{\partial Z}|}_{Bottom}=h_{b}\left(T-T_{a}\right)$$

The local temperature dramatically affects the heat transfer coefficient at the bottom face, which can be calculated using the following equation [35]:

(10) $$h_{b}=h_{b0}{\left(T-T_{a}\right)}^{0.25}$$

During the FSW process, the air (as the environment) surrounded the weld line and the top surface of the HDPE. Clearly, heat transfer models for convection and radiation were assumed for that area [36]:

(11) $$-k{\frac{\partial T}{\partial Z}|}_{Top}=Β\overset{`}{o}\left(T^{4}-T_{a}^{4}\right)+h_{t}\left(T-T_{a}\right)$$

**Figure 3:**
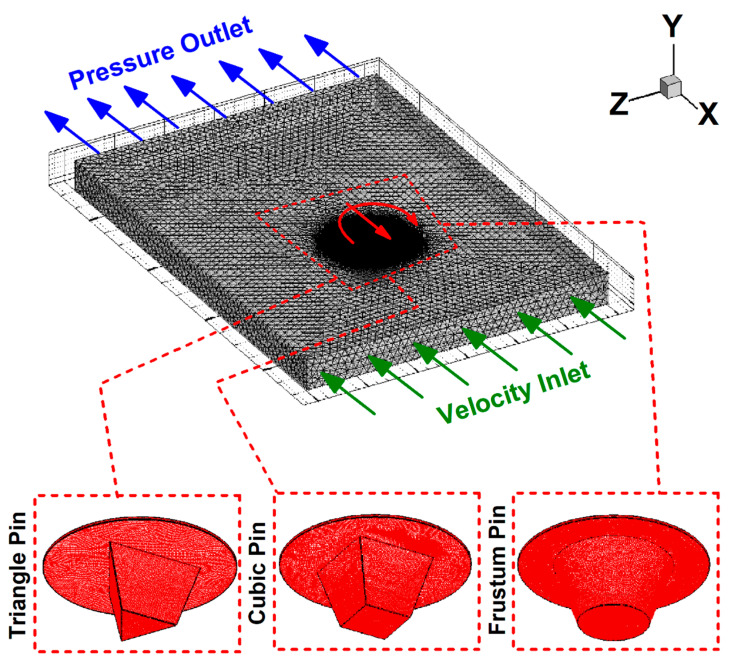
Meshed domain.

Three pin shapes were designed using frustum, triangular, and cubic geometries to study the effects of tool pin profiles. This simulation selected tetrahedral/hybrid elements with a T-grid shape for meshing the domain and used ANSYS Fluent software (ANSYS, Inc., Canonsburg, PA, USA) for equation solving. The simulation was tested by validating the results against experimental results. The overall errors of the simulation were less than 6% compared with experimental data. Figure 3 presents the meshed domain and pin profiles for the current work.

## 3. Materials and Methods

HDPE was selected as the joining material (WM). Transparent HDPE was selected because monitoring internal flow patterns was easier than opaque HDPE would be. The WM was provided from the local market, and the thermal properties and rheological properties of HDPE were examined in the laboratory according to ASTM C518, ASTM D7984, and ASTM E1461 test standards. After that, the raw HDPE sheet was cut into small pieces to prepare it for the FSW process.

**Figure 4:**
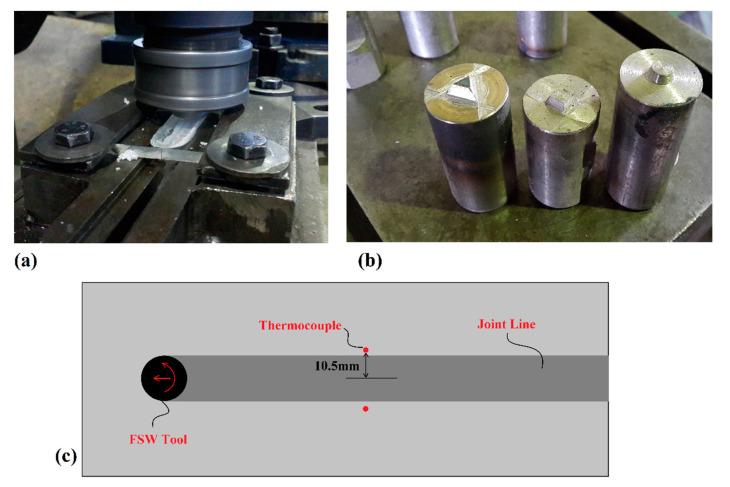
(**a**) FSW setup and joining process, (**b**) FSW tools, (**c**) Thermocouple placements.

The WM was fixed in a carbon-steel joining setup to be fixed during welding. A picture of the welding setup and WM during joining is presented in Figure 4a. For investigating the effects of pin profiles, three pin profiles, frustum pin (FPT), triangle pin (TPT), and cubic pin (CPT), were selected and tested. All tools were made from H13 steel with the same shoulder diameter; the schematic view and pin profile pictures are depicted in Figure 4b. For thermal history monitoring of the workpiece during the FSW process, J-type thermocouples (Omega Engineering, Norwalk, CT, USA) were used. To this end, the thermocouple locations were drilled at given positions and the thermocouples bonded inside the holes with Testors cement. The thermocouples were embedded into the HDPE sheets 10.5 mm from the joint line. The schematic view of the thermocouple locations is presented in Figure 4c. The thermocouples were attached to the top surface of the workpieces and thermal data were transferred to a convertor module (ADAM-4018+, Advantech, Irvine, CA, USA) to convert the signal from analog to digital. The digital data were transferred to a personal computer and monitored using LabView Software. During FSW, all process parameters were kept constant for all tools. The tools had 1200 rpm and 25 mm/min velocity. The tools’ tilt angle and plunge depth were 2° and 0.2 mm. After FSW, a video optical measurement machine (MUMA, 3D Family, Xinbei City, Taiwan) was used to investigate the surface flow rings and material flow analyses (surface and internal flow).

## 4. Results and Discussion

### 4.1. Heat Generation Rate

**Figure 5:**
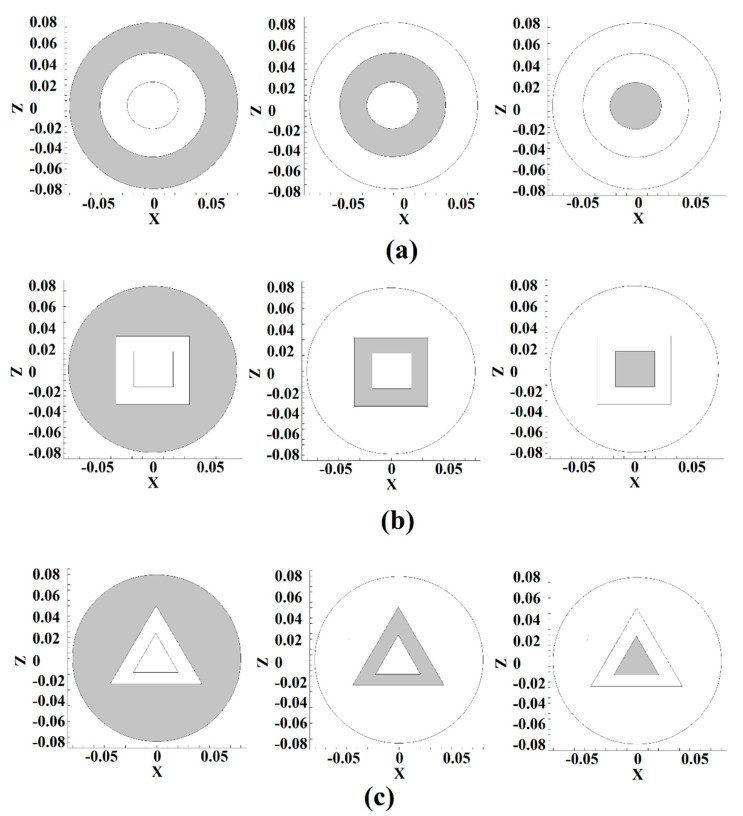
Shoulder, pin body, and pin bottom area for (**a**) FPT, (**b**) CPT, and (**c**) TPT.

During the FSW process, the joint surface between the workpiece and the tool is essential in generating frictional heat [37]. The friction at the interfaces makes frictional heat. For this reason, analysis of the contact surface between different tools for understanding thermal history is essential [38]. The contact area of various tools is depicted in Figure 5.

All tools have a shoulder–workpiece, pin body–workpiece, and pin bottom–-workpiece contact area. Figure 5a–c indicated different parts of the tool in contact with the base metal for FPT, CPT, and TPT pins, respectively. The geometrical analysis of the different tools is presented in Table 1.

The geometry analysis shows that the smallest contact area in the shoulder belongs to the FPT and the biggest belongs to the CPT. The shoulder–workpiece contact of the CPT was 65.27 mm^2^ more than the FPT pin. This trend changed at the pin body–workpiece and pin bottom–workpiece surfaces. The results showed that the CPT’s pin body–workpiece contact area was 52.05 mm^2^ less than the FPT. The total contact area between the FPT, TPT, and CPT were 324.77, 301.15, and 304.44 mm^2^, respectively. The simulation results indicated that the total heat generation in the joint line from the FPT, TPT, and CPT pins was 91, 94, and 96 °C., respectively The temperatures recorded by the thermocouples for the FPT, TPT, and CPT pins were 95, 99, and 103 °C, respectively.

### 4.2. Heat Distribution

**Figure 6:**
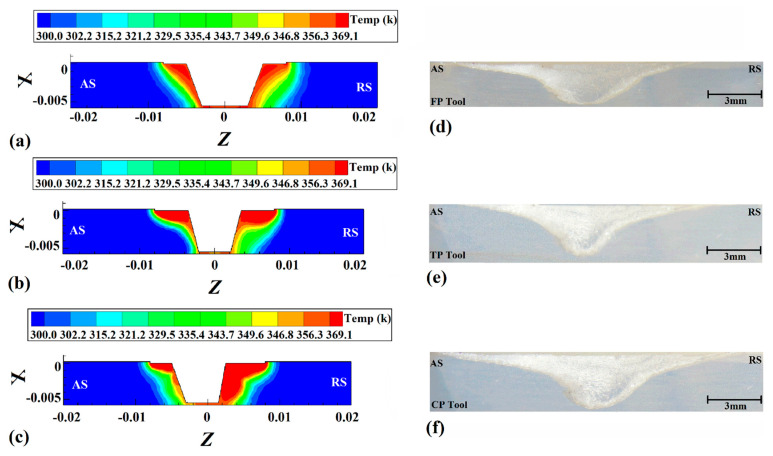
Simulation results in a cross-section view of internal heat flux with (**a**) FPT, (**b**) TPT and (**c**) CPT pins. Cross-section view of FSWed samples with (**d**) CPT, (**e**) CPT and (**f**) FPT pins.

The tool’s stirring action can determine heat flux by rotating the hot material [39]. According to the obtained results, the heat generation of the CPT was more than in other cases, and the heat generation in the FPT case was lowest. The cross-section views of internal heat flux results obtained via simulation are depicted in Figure 6. Figure 6a–c presents the internal heat flux of joints that were FSWed with a CPT, TPT, and FPT pin, respectively. The results revealed that the heat flux in the retreating side (RS) was more than that in the advancing side (AS). Due to the rotation of the FSW tool, the hot materials rotated from the AS to the RS, and this caused the heat concentration in the RS to be greater than in the AS. This behavior can be seen in all cases.

The tool rotation direction extrudes the materials from the advancing side and compresses the retreating side. This material dynamic caused the concentration of hot HDPE on the retreating side to be greater. The deposition of hot material on the retreating side led to more heat diffusion on that side of the joint line, so the heat flux in the RS was greater than in the AS. This behavior was related to the rotational direction of the tool, and was not related to the pin profile. Consequently, the heat flux in the retreating side for all joints was greater than in the advancing side.

The cross-section view of welded samples using CPT, TPT, and FPT pins is presented in Figure 6d, Figure 6e and Figure 6f, respectively. For a better analysis of internal material flow, the joint line was highly magnified. On the other hand, the simulation results were used without magnifications. Magnification of simulation results led to missing heat flux information, and low magnification of the stirring zone led to missing the internal flow results. There are reasons that the size (dimension window) of actual joints and simulation results are not presented as a 100% match. As the results show, the joint line in all samples consisted of a stirred zone (SZ), thermomechanical affected zone (TMAZ), and heat-affected zone (HAZ). The greater stirring action and heat generation caused the SZ in the CPT case to be bigger than in other cases, and the most petite SZ formed in the FPT joint. Consequently, the CPT case’s TMAZ and HAZ areas were bigger than for other cases.

**Figure 7:**
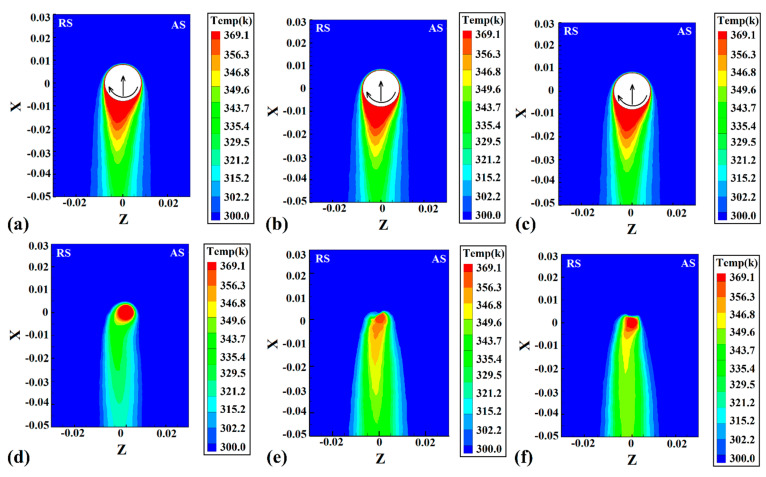
Simulation results of surface heat flow at joint FSWed by (**a**) FPT, (**b**) TPT, and (**c**) CPT pins. Simulation results of backplate heat flow at joint FSWed by (**d**) FPT, (**e**) TPT, and (**f**) CPT pins.

The surface heat flow on the workpieces’ top and bottom surfaces is depicted in Figure 7. As the results show, the surface heat flux was narrow in all cases. Due to the low heat transfer coefficient of the HDPE polymer used in this study, the surface heat flux is not comprehensive. The surface heat flux of joints that were FSWed by FPT, TPT, and CPT pins are depicted in Figure 7a, Figure 7b and Figure 7c, respectively. The difference is that the amount of generated heat from the CPT pin was higher and caused the hotter area seen on the surface. The difference between the bottom and top surfaces is that the bottom surface was in touch with the backing plate, and the heat transfer in this area was unlike the top surface. On the top surface, the HDPE polymer was in contact with air, and the heat transfer was much lower than on the bottom, which was in touch with steel.

The heat flux at the bottom of the joint welded by FPT, TPT, and CPT pins is depicted in Figure 7d, Figure 7e and Figure 7f, respectively. As seen, the hottest area was around the pin beneath. For this reason, the heat flux on the bottom side was more limited than on the top.

### 4.3. Internal Flow

The importance of studying the pin profile effects is because of their direct influence on the metal flow in the stirred zone (SZ) and the variation in the heat generated, which determines the mechanical properties of the welded joint [40]. The tool pin profile is the key point to getting a sound joint by affecting material flow and temperature. The design of the tool can be considered for avoiding weld thinning in metallic materials [41]. Due to the use of optimum process parameters during the joining of HDPE, weld thinning was not observed.

**Figure 8:**
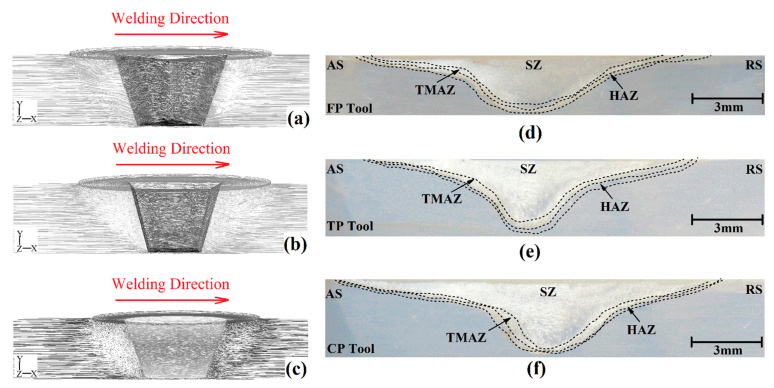
Simulation results of cross-section material flow at a joint FSWed by (**a**) FPT, (**b**) TPT, and (**c**) CPT pins. Cross-section view of a different joint area produced by (**d**) FPT, (**e**) TPT, and (**f**) CPT pins.

Figure 8 shows simulation results and a cross-section view of welded samples. Figure 8a–c indicates the material flow path simulation results for FPT, TPT, and CPT pins. From obtained results, we can see that the internal stirring action of the FSW tool increased by increasing the number of edges of the pin. It means that the stirring action of the CPT is greater than other pins, and the FPT’s stirring action is lower than other tools. More stirring action resulted from higher thermomechanical action in the stirring zone created by the FSW tool. Material mixing, increasing material extrusion from the advancing side to the retreating side, and formation of a bigger thermomechanically affected zone were results of higher stirring action. Due to the greater stirring action of the tool, the joint line size changed [42]. The size of the joint line that was FSWed by the CPT was more than in other cases. A cross-section view of FSWed samples welded by FPT, TPT, and CPT pins is depicted in Figure 8d, Figure 8e and Figure 8f, respectively. As discussed before, the SZ, TMAZ, and HAZ formed in all joints. The results revealed that the sizes of the SZ, TMAZ and HAZ of welded samples were not the same. These differences resulted from the tool’s generated heat and stirring action [43]. The thickest HAZ and TMAZ were formed by the CPT, and thinner HAZ and TMAZ were formed by the FPT, respectively.

**Figure 9:**
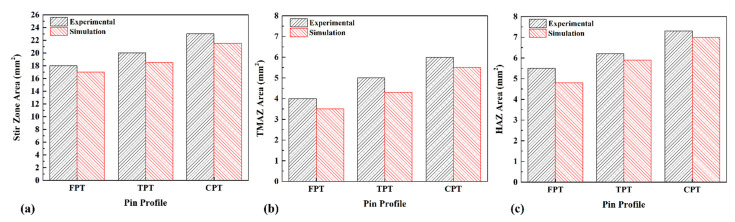
Comparison between dimensions of (**a**) stirred zone, (**b**) TMAZ, and (**c**) HAZ areas predicted by simulation and measured experimentally.

The geometrical assessment of different joint lines is depicted in Figure 9. The comparison between the predicted size of the SZ and experimental results is presented in Figure 9a. The results from experimental tests indicated that the size of the SZ increased from18 mm^2^ (FPT) to 23 mm^2^ for the CPT. The simulation results show that the SZ’s size for the FPT increased from17 mm^2^ to 21 mm^2^ for the CPT. The TMAZ area measurement revealed increasing stirring action led to the formation of a too-large TMAZ in a joint line. Figure 9b depicts the results of the size of TMAZ at different joints. The experimental results of TMAZ size in the joints that FPT, TPT, and CPT pins welded were 4 mm^2^, 5 mm^2^, and 6 mm^2^, respectively. The simulation results of TMAZ size in the joint welded by FPT, TPT, and CPT pins were 3.5 mm^2^, 4.5 mm^2^, and 5.5 mm^2^, respectively. Increasing heat generation increased heat diffusion growth and size of the HAZ. Figure 9c depicts a comparison of the size results for HAZ at different joints. The experimental results of HAZ size in the joint welded by FPT, TPT, and CPT pins were 5.5 mm^2^, 6.5 mm^2^, and 7.5 mm^2^, respectively. The HAZ size simulation results in the joint welded by FPT, TPT, and CPT pins were 5.3 mm^2^, 6.1 mm^2^, and 7.2 mm^2^, respectively.

### 4.4. Joint Formation Mechanism

**Figure 10:**
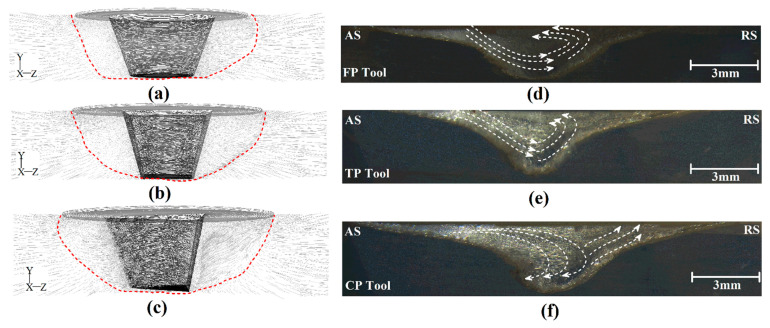
Internal material flow around (**a**) FPT, (**b**) TPT, and (**c**) CPT pins. Internal flow path of joints welded by (**d**) FPT, (**e**) TPT, and (**f**) CPT pins.

Internal flow can analyze the joining mechanism during the FSW of polymeric materials [44]. As discussed above, the internal flow of materials can be analyzed using simulation results. Figure 10a–c shows the cross-section view of the stirring areas made by FPT, TPT, and CPT pins, respectively. The simulation results indicated that the edges of the pin changed the internal flow. The internal flow of joints that were FSWed by FPT, TPT, and CPT pins are depicted in Figure 10d, Figure 10e and Figure 10f, respectively. The joining mechanism consisted of the uniform mixing of materials extruded from the AS to the RS. In the middle of the SZ, the plasticized polymer’s mixing pattern can determine the joint’s quality [45]. In the FPT-welded sample, the tool extruded materials from the AS to the RS, and a circular flow pattern can be seen in the SZ. In the TPT case, the materials were extruded to the middle and lower areas of the SZ and circulated in the middle of the SZ. In the case of the CPT pin, the flow pattern was non-uniform. The material was extruded from the AS to the center of the SZ. Some of the material reverted to the AS, and the rest was extruded to the RS. The analysis of experimental results revealed that during FSW of polymeric materials, the edges of the pin increased the irregular mixing pattern in the SZ. This flow pattern caused air bubbles to be trapped during the joining process and decreased the final quality of the joint.

Four points in the stir zone were selected for a better analysis of material velocity. The selected point’s positions from the top view are depicted in Table 2. One point was selected at the exterior area of the tool shoulder (point (a)), one point was selected at the interface of shoulder and pin (point (b)), one point was selected at the pin bottom (point (c)), and the last point selected at pin edges at the interface of the pin–shoulder surface (point (d)). The results of the HDPE velocity simulation are presented in Table 2. From the obtained results, we can see that the highest material velocity was on point (a) for all cases. The high distance between point (a) and the tool axis caused high torque in point (a), and material velocity at this point became high.

On the other hand, the lowest material velocity was produced in point (c). Similarly, the material velocity at this point was low due to the distance between the tool axis and point (c). The results of points (b) and (d) for the effects of pin edges are compared. The simulation results showed that there was not any difference between points (b) and (d) for the FPT because the pin does not have an edge. Nevertheless, the material velocity at the edges of the TPT and CPT was 25% more than the flat part of the pin.

**Figure 11:**
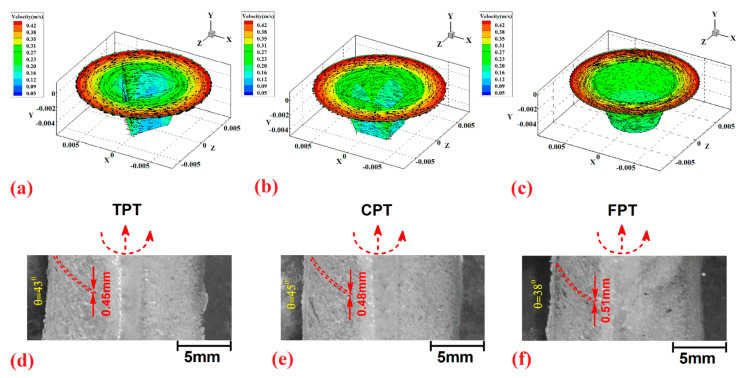
Material velocity inside the stirring zone for joints FSWed by (**a**) TPT, (**b**) CPT, and (**c**) FPT pins. Surface flow of joints FSWed by (**d**) TPT, (**e**) CPT, and (**f**) FPT pins.

The simulated material velocities in the SZ for the TPT, CPT, and FPT pins are depicted in Figure 11a–c. The obtained results indicate that the velocity of the materials increased with the increase in edges of the FSW tool pin. According to the results of the simulation, the velocity of the materials for the TPT, CPT, and FPT pins were 0.41 m/s, 0.42 m/s, and 0.4 m/s, respectively. The results indicated that the maximum material velocity was from the CPT, and the minimum material velocity was predicted for the FPT. The material velocity in the SZ affects surface material flow. The top view of the surface flow of joints welded by TPT, CPT, and FPT pins is depicted in Figure 11d, Figure 11e and Figure 11f, respectively. The over-stirring action of the CPT caused the formation of unequal flow rings at the surface of the joint line. The results revealed that the distance between flow rings at the surface of the joint line was 0.45 mm, 0.48 mm, and 0.51 mm for TPT, CPT, and FPT joints, respectively. The angle of flow rings in joints that were FSWed with TPT, CPT, and FPT pins were 43°, 45°, and 38°, respectively.

## 5. Conclusions

This research successfully used a thermomechanical simulation of HDPE with a CFD approach to analyze the influence of FSW tool pin profiles on heat generation and material flow, where material experiments were used to validate the simulation’s findings. The study’s main results are presented below:

1. Due to the higher contact surface, maximum heat was generated in the CPT case, and the lowest was generated in the FPT sample. According to the results, the generated heat in the joint that was FSWed with a CPT (96 °C) was ~6% more than that using an FPT (91 °C).
2. Higher heat generation in the CPT sample led to greater heat flux inside and on the surface of the joint line, which caused a bigger SZ to form in the CPT sample compared to the others. The simulation results indicate that the SZ size of samples that were FSWed with FPT, TPT, and CPT pins were 17 mm^2^, 19 mm^2^, and 21 mm^2^, respectively, which is around three times the corresponding values in the HAZ.
3. The simulated velocity of the materials inside of the SZ increased with the number of the pin’s edges. This phenomenon led to an irregular internal flow of the HDPE, which increased over-stirring and air trapped inside the joint line.

## Figures and Tables

**Table 1:** Comparison between the area of tools and heat generation rate.

	Area (mm^2^)			Simulation Results for Generated Heat (°C)			Maximum Recorded Temperature (°C)		
	FPT	TPT	CPT	FPT	TPT	CPT	FPT	TPT	CPT
Shoulder	141.73	182.47	207.7	42	48	59	-	-	-
Pin Body	140.61	114.12	88.56	40	39	31	-	-	-
Pin Bottom	42.79	14.95	7.17	9	7	6	-	-	-
Total	324.77	301.54	303.44	91	94	96	95	99	103

**Table 2:** Analysis of material velocity.

Tool Type	Velocity Point (a)	Velocity Point (b)	Velocity Point (c)	Velocity Point (d)
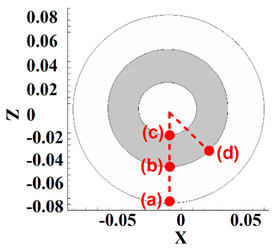	0.4 m/s	0.15 m/s	0.1 m/s	0.15 m/s
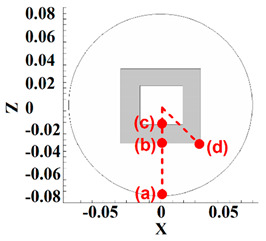	0.42 m/s	0.2 m/s	0.12 m/s	0.25 m/s
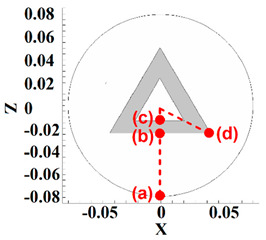	0.41 m/s	0.18 m/s	0.11 m/s	0.22 m/s

## Data Availability

Not applicable.

## References

[1] Al-Sabur R.K., Jassim A.K. Friction Stir Spot Welding Applied to Weld Dissimilar Metals of AA1100 Al-Alloy and C11000 Copper. Proceedings of the IOP Conference Series: Materials Science and Engineering, 2nd International Conference on Advancements in Aeromechanical Materials for Manufacturing.

[2] Meng X., Huang Y., Cao J., Shen J., dos Santos J.F. (2021). Recent Progress on Control Strategies for Inherent Issues in Friction Stir Welding. Prog. Mater. Sci..

[3] Al-Sabur R. (2021). Tensile Strength Prediction of Aluminium Alloys Welded by FSW Using Response Surface Methodology-Comparative Review. Mater. Today Proc..

[4] Asmare A., Al-Sabur R., Messele E. (2020). Experimental Investigation of Friction Stir Welding on 6061-T6 Aluminum Alloy Using Taguchi-Based GRA. Metals.

[5] Mugada K.K., Adepu K. (2018). Influence of Ridges Shoulder with Polygonal Pins on Material Flow and Friction Stir Weld Characteristics of 6082 Aluminum Alloy. J. Manuf. Process..

[6] Elangovan K., Balasubramanian V., Valliappan M. (2008). Effect of Tool Pin Profile and Tool Rotational Speed on Mechanical Properties of Friction Stir Welded AA6061 Aluminium Alloy. Mater. Manuf. Process..

[7] Biswas P., Kumar D.A., Mandal N.R. (2012). Friction Stir Welding of Aluminum Alloy with Varying Tool Geometry and Process Parameters. Proc. Inst. Mech. Eng. B J. Eng. Manuf..

[8] Khodaverdizadeh H., Heidarzadeh A., Saeid T. (2013). Effect of Tool Pin Profile on Microstructure and Mechanical Properties of Friction Stir Welded Pure Copper Joints. Mater. Des..

[9] Imam M., Biswas K., Racherla V. (2013). Effect of Weld Morphology on Mechanical Response and Failure of Friction Stir Welds in a Naturally Aged Aluminium Alloy. Mater. Des..

[10] Mehta M., De A., DebRoy T. (2014). Material Adhesion and Stresses on Friction Stir Welding Tool Pins. Sci. Technol. Weld. Join..

[11] Mehta M., Reddy G.M., Rao A.V., De A. (2015). Numerical Modeling of Friction Stir Welding Using the Tools with Polygonal Pins. Def. Technol..

[12] Venkata Rao C., Madhusudhan Reddy G., Srinivasa Rao K. (2015). Microstructure and Pitting Corrosion Resistance of AA2219 Al–Cu Alloy Friction Stir Welds–Effect of Tool Profile. Def. Technol..

[13] Amirafshar A., Pouraliakbar H. (2015). Effect of Tool Pin Design on the Microstructural Evolutions and Tribological Characteristics of Friction Stir Processed Structural Steel. Measurement.

[14] Aghajani Derazkola H., Kordani N., Mohammadi Abokheili R. (2022). Investigation of Joining Mechanism of Electrical-Assist Friction Stir Joining between Polyethylene (PE) and 316 Stainless Steel. Arch. Civ. Mech. Eng..

[15] Lambiase F., Derazkola H.A., Simchi A. (2020). Friction Stirwelding and Friction Spot Stir Welding Processes of Polymers-State of the Art. Materials.

[16] Dashatan S.H., Azdast T., Ahmadi S.R., Bagheri A. (2013). Friction Stir Spot Welding of Dissimilar Polymethyl Methacrylate and Acrylonitrile Butadiene Styrene Sheets. Mater. Des..

[17] Huang Y., Meng X., Xie Y., Wan L., Lv Z., Cao J., Feng J. (2018). Friction Stir Welding/Processing of Polymers and Polymer Matrix Composites. Compos. Part A Appl. Sci. Manuf..

[18] Panneerselvam K., Lenin K. (2014). Joining of Nylon 6 Plate by Friction Stir Welding Process Using Threaded Pin Profile. Mater. Des..

[19] Lambiase F., Paoletti A., di Ilio A. (2015). Mechanical Behaviour of Friction Stir Spot Welds of Polycarbonate Sheets. Int. J. Adv. Manuf. Technol..

[20] Lambiase F., Paoletti A., di Ilio A. (2017). Effect of Tool Geometry on Mechanical Behavior of Friction Stir Spot Welds of Polycarbonate Sheets. Int. J. Adv. Manuf. Technol..

[21] Al-Sabur R., Khalaf H.I., Świerczyńska A., Rogalski G., Derazkola H.A. (2022). Effects of Noncontact Shoulder Tool Velocities on Friction Stir Joining of Polyamide 6 (PA6). Materials.

[22] Gao J., Cui X., Liu C., Shen Y. (2017). Application and Exploration of Friction Stir Welding/Processing in Plastics Industry. Mater. Sci. Technol..

[23] Rezaee Hajideh M., Farahani M., Alavi S.A.D., Molla Ramezani N. (2017). Investigation on the Effects of Tool Geometry on the Microstructure and the Mechanical Properties of Dissimilar Friction Stir Welded Polyethylene and Polypropylene Sheets. J. Manuf. Process..

[24] Sahu S.K., Mishra D., Mahto R.P., Sharma V.M., Pal S.K., Pal K., Banerjee S., Dash P. (2018). Friction Stir Welding of Polypropylene Sheet. Eng. Sci. Technol. Int. J..

[25] Sadeghian N., Besharati Givi M.K. (2015). Experimental Optimization of the Mechanical Properties of Friction Stir Welded Acrylonitrile Butadiene Styrene Sheets. Mater. Des..

[26] Aghajani Derazkola H., Simchi A. (2018). Experimental and Thermomechanical Analysis of the Effect of Tool Pin Profile on the Friction Stir Welding of Poly(Methyl Methacrylate) Sheets. J. Manuf. Process..

[27] Khodabakhshi F., Derazkola H.A., Gerlich A.P. (2020). Monte Carlo Simulation of Grain Refinement during Friction Stir Processing. J. Mater. Sci..

[28] Khalaf H.I., Al-sabur R., Abdullah M.E., Kubit A., Derazkola H.A. (2022). Effects of Underwater Friction Stir Welding Heat Generation on Residual Stress of AA6068-T6 Aluminum Alloy. Materials.

[29] Talebizadehsardari P., Musharavati F., Khan A., Sebaey T.A., Eyvaziana A., Derazkola H.A. (2021). Underwater Friction Stir Welding of Al-Mg Alloy: Thermo-Mechanical Modeling and Validation. Mater. Today Commun..

[30] Aghajani Derazkola H., Garcia E., Elyasi M. (2021). Underwater Friction Stir Welding of PC: Experimental Study and Thermo-Mechanical Modelling. J. Manuf. Process..

[31] Eyvazian A., Hamouda A.M., Aghajani Derazkola H., Elyasi M. (2020). Study on the Effects of Tool Tile Angle, Offset and Plunge Depth on Friction Stir Welding of Poly(Methyl Methacrylate) T-Joint. Proc. Inst. Mech. Eng. B J. Eng. Manuf..

[32] Elyasi M., Derazkola H.A. (2018). Experimental and Thermomechanical Study on FSW of PMMA Polymer T-Joint. Int. J. Adv. Manuf. Technol..

[33] Derazkola H.A., Eyvazian A., Simchi A. (2020). Modeling and Experimental Validation of Material Flow during FSW of Polycarbonate. Mater. Today Commun..

[34] Memon S., Murillo-Marrodán A., Lankarani H.M., Aghajani Derazkola H. (2021). Analysis of Friction Stir Welding Tool Offset on the Bonding and Properties of al–Mg–Si Alloy t-Joints. Materials.

[35] Mirzaei M.H., Asadi P., Fazli A. (2020). Effect of Tool Pin Profile on Material Flow in Double Shoulder Friction Stir Welding of AZ91 Magnesium Alloy. Int. J. Mech. Sci..

[36] Sevvel P., Dhanesh Babu S.D., Senthil Kumar R. (2020). Peak Temperature Correlation and Temperature Distribution during Joining of AZ80A Mg Alloy by FSW-A Numerical and Experimental Investigation. Stroj. Vestn. J. Mech. Eng..

[37] Derazkola H.A., MohammadiAbokheili R., Kordani N., Garcia E., Murillo-Marrodán A. (2022). Evaluation of Nanocomposite Structure Printed by Solid-State Additive Manufacturing. CIRP J. Manuf. Sci. Technol..

[38] Hernández C.A., Ferrer V.H., Mancilla J.E., Martínez L.C. (2017). Three-Dimensional Numerical Modeling of the Friction Stir Welding of Dissimilar Steels. Int. J. Adv. Manuf. Technol..

[39] Bahedh A.S., Mishra A., Al-Sabur R., Jassim A.K. (2022). Machine Learning Algorithms for Prediction of Penetration Depth and Geometrical Analysis of Weld in Friction Stir Spot Welding Process. Metall. Res. Technol..

[40] Guan M., Wang Y., Huang Y., Liu X., Meng X., Xie Y., Li J. (2019). Non-Weld-Thinning Friction Stir Welding. Mater. Lett..

[41] Ma X., Xie Y., Meng X., Chen H., Wang F., Jiang Y., Wan L., Huang Y. (2021). Stepped-Shoulder Friction Stir Welding to Alleviate Weld Thinning for Dissimilar AA2195-T8/AA2219-T6 Alloys. Sci. Technol. Weld. Join..

[42] Memon S., Fydrych D., Fernandez A.C., Derazkola H.A., Derazkola H.A. (2021). Effects of Fsw Tool Plunge Depth on Properties of an Al-Mg-Si Alloy t-Joint: Thermomechanical Modeling and Experimental Evaluation. Materials.

[43] Aghajani Derazkola H., Kordani N., Aghajani Derazkola H. (2021). Effects of Friction Stir Welding Tool Tilt Angle on Properties of Al-Mg-Si Alloy T-Joint. CIRP J. Manuf. Sci. Technol..

[44] Mendes N., Loureiro A., Martins C., Neto P., Pires J.N. (2014). Effect of Friction Stir Welding Parameters on Morphology and Strength of Acrylonitrile Butadiene Styrene Plate Welds. Mater. Des..

[45] Aghajani Derazkola H., Simchi A. (2020). Processing and Characterizations of Polycarbonate/Alumina Nanocomposites by Additive Powder Fed Friction Stir Processing. Thin Walled Struct..

